# 330. BioFire ® FilmArray® Pneumonia Panel (PN) Results Compared to Standard Microbiologic Testing and Clinical Adjudication in Adults Hospitalized with Respiratory Illness.

**DOI:** 10.1093/ofid/ofac492.408

**Published:** 2022-12-15

**Authors:** Ann R Falsey, Angela R Branche, Daniel P Croft, Maria A Formica, Michael Peasley, Jyotimayee Lenka, Naman Sharma, Edward E Walsh

**Affiliations:** University of Rochester School of Medicine and Dentistry, Rochester, NY; Rochester Regional Health, Rochester, NY, USA, Rochester, New York; University of Rochester, Rochester, New York; University of Rochester Medical Center, Rochester, New York; Rochester Regional Health, Rochester, New York; University of Rochester, Rochester, New York; University of Arizona Tucson, Tucson, Arizona; University of Massachusetts - Baystate Medical Center, Enfield, Connecticut; University of Rochester, Rochester Regional Health, Rochester, New York

## Abstract

**Background:**

Inability to define microbial etiology of lower respiratory tract infection (LRTI) leads to unnecessary antibiotic use. Though multiplex PCR improves viral detection, bacterial LRTI diagnosis remains problematic. We evaluated the clinical utility of BioFire ® FilmArray ® Pneumonia (PN) Panel which detects 8 viruses, 3 atypical and 15 pathogenic bacteria by semi-quantitative PCR to inform bacterial diagnosis.

**Methods:**

As part of a study to distinguish bacterial from non-bacterial LRTI in hospitalized adults, clinical, laboratory, and X-ray data were collected. Adequate sputa (< 10 Epithelial & >25 PMNs) processed by Gram stain and culture were tested with FilmArray® PN. Microbial LRTI etiology (viral alone, bacterial with or without concomitant viruses, or indeterminate) was adjudicated by a four physician panel using all clinical data except bacterial PCR results. Bacterial PCR was compared to Gram stain and culture and clinical adjudication.

**Results:**

From 737 illnesses evaluated, 423 sputa were collected and 201 deemed adequate and were tested with FilmArray® PN. Most common discharge diagnoses were pneumonia (24%), AECOPD (21%), viral illness (13%) and asthma exacerbations (9%). FilmArray® PN detected 155 typical bacteria, 9 atypical and 101 viruses. Most bacterial detections were monomicrobial (58%) often with concomitant viruses (43%). Compared to Gram stain and culture, FilmArray® PN detected more bacterial pathogens and was less affected by antibiotics. (Figure) Cases were adjudicated as viral Alone (37), bacterial (93) and indeterminate (71). Bacteria were detected by PCR in 41% of viral and 96% of bacterial cases, p=0.0001 and 76% of indeterminate cases. In cases with no bacteria detected by PCR, only 4 (9%) were adjudicated as bacterial; all deemed caused by anaerobic bacteria which are not included in the PCR panel. Comparing bacterial vs. non-bacterial (Viral + Indeterminate), FilmArray® PN bacterial PCR had 96% sensitivity, 36% specificity, 56% positive predictive value and 91% negative predictive value. Finally, sputum PCR detected 4 mycoplasma and 56 viral infections missed by standard of care testing.

**Figure:**
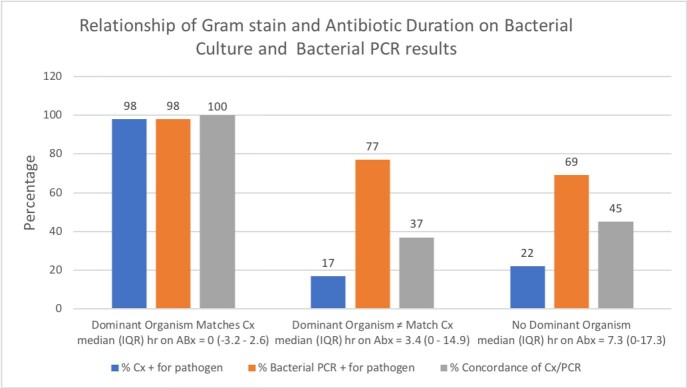
Relationship of Gram Stain and Time on Antibiotics to Bacterial Culture and Bacterial PCR Results

**Conclusion:**

Multiplex PCR testing of sputa for bacteria is useful to rule out bacterial infection with added value to detect viruses and atypical bacteria.

**Disclosures:**

**Ann R. Falsey, MD**, BioFire Diagnostics: Grant/Research Support|Janssen: Grant/Research Support|Merck, Sharp and Dohme: Grant/Research Support|Novavax: Advisor/Consultant|Pfizer: Grant/Research Support **Angela R. Branche, MD**, Cyanvac: Grant/Research Support|GSK: Advisor/Consultant|Janssen: Advisor/Consultant|Merck: Expert Testimony|Pfizer: Grant/Research Support **Edward E. Walsh, MD**, Janssen: Advisor/Consultant|Janssen: Grant/Research Support|Janssen: Honoraria|Merck: Advisor/Consultant|Merck: Grant/Research Support|Pfizer: Advisor/Consultant|Pfizer: Grant/Research Support.

